# Impact of Accumulated Institutional Experience on Outcomes of Cytoreductive Surgery with Intraperitoneal Chemotherapy for Colorectal and Appendiceal Peritoneal Metastases: Early Versus Later Cohort Analysis

**DOI:** 10.3390/cancers18091416

**Published:** 2026-04-29

**Authors:** Jung Wook Suh, Hyelim Kang, Hwan Namgung, Jae Won Jo, Sung Chul Lee, Dong-Guk Park

**Affiliations:** Department of Surgery, Dankook University Hospital, Cheonan-si 31116, Republic of Korea; jickgack@dkuh.co.kr (J.W.S.); chsonon@dkuh.co.kr (J.W.J.); leesc292513@dankook.ac.kr (S.C.L.); dkpark@dankook.ac.kr (D.-G.P.)

**Keywords:** colorectal cancer, peritoneal carcinomatosis, cytoreductive surgery, HIPEC, prognosis, treatment outcome

## Abstract

**Background:** Cytoreductive surgery (CRS) combined with intraperitoneal chemotherapy (IPC) is an established treatment for select patients with colorectal peritoneal metastases. However, the impact of evolving treatment strategies and accumulating institutional experience on oncological outcomes remains poorly understood. This study aimed to characterise temporal changes in treatment approaches over a decade at a single high-volume centre and evaluate their association with outcomes by comparing early and later patient cohorts. **Methods:** A total of 160 patients who underwent CRS with IPC for colorectal peritoneal metastases between 2011 and 2019 were retrospectively analysed and categorised into early (2011–2013, *n* = 42) and late (2014–2019, *n* = 118) cohorts. Overall survival (OS) and disease-free survival (DFS) were compared between the groups. Prognostic factors were assessed using Cox proportional hazard regression models. **Results:** The later cohort demonstrated significantly improved OS compared to the early cohort (median OS, 25.1 vs. 13.8 months; 5-year OS, 25.9% vs. 11.9%; log-rank *p* = 0.015). DFS showed a non-significant trend toward improvement (*p* = 0.176). In the multivariate analysis, cohort period (HR 0.63, *p* = 0.029), completeness of cytoreduction (HR 4.51, *p* < 0.001), tumour location, preoperative CEA level, and receipt of preoperative systemic chemotherapy were independently associated with OS. **Conclusions:** These findings suggest that accumulated institutional experience and changes in treatment strategies may be associated with improved OS in patients undergoing CRS with IPC for colorectal peritoneal metastases.

## Simple Summary

Cytoreductive surgery combined with intraperitoneal chemotherapy represents a treatment strategy that can improve survival in selected patients with colorectal and appendiceal cancers with peritoneal metastases. However, given the complexity of this approach, optimal outcomes are most likely to be achieved when it is performed at experienced, specialized centers. In this study, we analyzed 160 patients treated over a 10-year period at a single specialized center, comparing outcomes between an early and a later cohort. We observed a significant improvement in overall survival over time, in parallel with the accumulation of institutional experience and the evolution of treatment strategies. In addition, achieving complete cytoreduction and timely referral to a specialized center were identified as key factors associated with improved survival. These findings underscore the importance of delivering appropriate, specialized care at the right time for patients with colorectal and appendiceal peritoneal metastases.

## 1. Introduction

Colorectal cancer is one of the most prevalent malignancies worldwide, ranking third among all cancers, with over 1.9 million new cases diagnosed in 2022 [1,2]. Among patients with colorectal cancer, peritoneal metastasis is the third most common site of metastasis after hepatic and pulmonary metastases [3,4], and approximately 4% of patients present with isolated peritoneal dissemination [5]. Compared to other metastatic sites, peritoneal metastases respond poorly to systemic chemotherapy, with a median survival of only 10–18 months despite active medical treatment [4,6,7].

In selected patients with peritoneal metastases from various primary tumours, including appendiceal, gastric, and ovarian cancers, cytoreductive surgery (CRS) combined with intraperitoneal chemotherapy (IPC) has demonstrated improved survival outcomes compared to systemic chemotherapy alone [8,9]. In the context of colorectal peritoneal metastases, the landmark randomised PRODIGE 7 trial [10] established CRS as the cornerstone treatment by confirming its significant survival benefits. Notably, the trial reported no additional survival advantage with short-duration (30-min) oxaliplatin-based hyperthermic intraperitoneal chemotherapy (HIPEC); however, this finding should be interpreted with caution as it reflects a protocol-specific limitation rather than a class effect of HIPEC. Indeed, accumulating evidence from cohort studies indicates that mitomycin C–based HIPEC, particularly when administered with an optimised duration and technique, remains associated with favourable oncological outcomes [11].

Therefore, the management of colorectal peritoneal metastases requires a comprehensive multidisciplinary framework incorporating CRS, HIPEC, and perioperative systemic therapy [12]. Furthermore, high-volume centres with substantial institutional experience are uniquely positioned to refine patient selection, standardise surgical quality, and implement evolving therapeutic strategies, thereby achieving superior survival outcomes [13,14,15,16].

This study aimed to describe the evolution of treatment strategies and to evaluate their impact on oncological outcomes in patients undergoing CRS with IPC for colorectal peritoneal metastases at a single institution over a decade, with particular focus on comparing outcomes between early and later patient cohorts as institutional experience accumulated.

## 2. Materials and Methods

### 2.1. Study Design and Patient Selection

This retrospective cohort study was conducted at the Dankook University Hospital, a tertiary referral centre that performs approximately 50 CRS with IPC procedures annually for peritoneal surface malignancies. Patients who underwent CRS with IPC for colorectal and appendiceal peritoneal metastases between January 2011 and December 2019 were identified from the institutional surgical databases and medical records. This study was approved by the Institutional Review Board of Dankook University Hospital (No. 2025-11-003) and conducted in accordance with the Declaration of Helsinki. The requirement for informed consent was waived due to the retrospective nature of the study.

**Figure 1:**
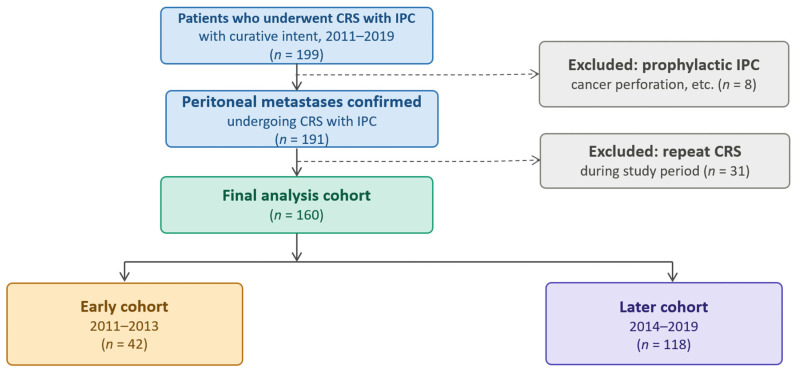
Patient selection flowchart.

As shown in Figure 1, a total of 199 patients were initially identified. Patients without macroscopic peritoneal disease who underwent prophylactic IPC (*n* = 8) and those who underwent repeat CRS during the study period (*n* = 31) were excluded. The final analysis cohort comprised 160 patients.

The patients were divided into two cohorts based on the year of surgery, reflecting a transition in the institutional treatment strategy: an early cohort (2011–2013, *n* = 42) and a later cohort (2014–2019, *n* = 118). This time point corresponds to the institutional adoption of HIPEC as the primary IPC modality, replacing early postoperative intraperitoneal chemotherapy (EPIC).

### 2.2. Surgical Technique, IPC Protocol, and Follow-Up

Prior to surgery, all patients underwent a comprehensive preoperative evaluation, including measurement of serum tumour markers (carcinoembryonic antigen [CEA] and cancer antigen [CA] 19-9), contrast-enhanced computed tomography (CT) of the chest and abdomen, and positron emission tomography–CT (PET-CT) to evaluate the extent of peritoneal involvement and distant metastases. Magnetic resonance imaging (MRI) was performed when hepatic or pelvic disease was suspected. Surgical candidacy was determined based on a thorough assessment of the medical history, functional status, and cardiopulmonary reserve. Patients were deemed ineligible for surgery if they had unresectable extraperitoneal disease, an Eastern Cooperative Oncology Group (ECOG) performance status exceeding 2, or if the patient or their guardian declined the procedure.

CRS was performed by a dedicated two-surgeon team with a combined institutional volume exceeding 50 cases per year. Complete macroscopic clearance of all visible tumours was the primary surgical objective. At the time of laparotomy, the extent of peritoneal disease was quantified using the Peritoneal Cancer Index (PCI), a standardised intraoperative scoring tool that evaluates 13 abdominal regions on a scale of 0–3, with a maximum possible score of 39. Peritoneal and visceral resections were performed using the systematic peritonectomy approach established by Sugarbaker [17]. Cytoreductive adequacy was defined as a Completeness of Cytoreduction (CCR) score of 0 (absence of a visible residual tumour) or 1 (residual nodules not exceeding 2.5 mm in diameter).

The IPC regimen employed after CRS reflected the evolution of institutional practices across the study period. During the early cohort period (2011–2013), EPIC was the dominant form of IPC. This involved intraperitoneal instillation of mitomycin C beginning on the first postoperative day, with 5-fluorouracil administered on days 2–5; the agents were retained within the peritoneal cavity for 23 h and drained for 1 h each day. As institutional experience grew, HIPEC replaced EPIC as the preferred modality in the later cohort (2014–2019). HIPEC was administered intraoperatively at the conclusion of CRS under general anaesthesia via the closed-abdomen technique using a Belmont Hyperthermia Pump (Belmont Instrument Corp., Billerica, MA, USA). Perfusion consisted of mitomycin C at a cumulative dose of 40 mg, with an initial 30 mg delivered over the first 60 min and a supplemental 10 mg administered over the final 30 min, circulated at an inflow temperature of 42–43 °C for a total duration of 90 min, previously reported protocols [18,19]. In selected patients in the later cohort, EPIC was administered after HIPEC. Regardless of the IPC modality, all patients were transferred to the intensive care unit for a minimum of 24 h for postoperative monitoring.

Structured postoperative surveillance was performed for all patients. Clinical assessments and serum CEA and CA 19-9 measurements were scheduled at 3-month intervals during the first 2 postoperative years and transitioned to 6-month intervals for up to 10 years thereafter. Cross-sectional imaging of the chest, abdomen, and pelvis was performed every 3–6 months in the first 2 years and every 6–12 months subsequently. In cases where disease recurrence was clinically suspected, supplementary imaging modalities, such as hepatic or pelvic MRI or PET/CT, were requested at the physician’s discretion, and the follow-up interval was adjusted accordingly.

### 2.3. Data Collection and Study Outcomes

Clinical and oncological data were systematically extracted from institutional medical records, and the survival status was verified using the National Vital Statistics Registry. The variables collected included patient demographics (age and sex), operative findings (primary tumour location, disease presentation timing, extent of peritonectomy, PCI grade, CCR score, and IPC modality), preoperative laboratory values (CEA and CA 19-9 levels and history of prior systemic chemotherapy), tumour histology, occurrence and severity of complications arising within 30 days of surgery as graded by the Clavien–Dindo system [20], duration of hospital stay, receipt of adjuvant chemotherapy, and long-term survival data.

The primary tumour location was designated as one of the four anatomical categories: appendix, right colon (encompassing the caecum, ascending colon, and transverse colon), left colon (descending colon and sigmoid colon), or rectum (defined as tumours located within 15 cm of the anal verge). The temporal relationship between the primary tumour and peritoneal metastasis was characterised as synchronous when peritoneal involvement was identified concurrently with or within 6 months of the primary diagnosis and as metachronous when detected beyond this interval. For the purposes of this study, preoperative systemic chemotherapy refers to any palliative intent chemotherapy administered before CRS, irrespective of the drug regimen, treatment line, or administering institution. With respect to tumour histology, the cases were grouped into two broad categories: well- or moderately differentiated adenocarcinoma (WD/MD) and aggressive subtypes comprising poorly differentiated adenocarcinoma, signet-ring cell carcinoma, and mucinous carcinoma (PD/SRC/MUC).

To facilitate subgroup comparisons, the intraoperatively determined PCI scores were stratified into three categories: low (0–10), intermediate (11–20), and high (>20) [21]. Similarly, CCR scores were consolidated into a binary classification for analytical purposes: favourable (CCR0–1, indicating complete cytoreduction) and unfavourable (CCR2–3, reflecting incomplete cytoreduction) [22].

The primary study endpoint was overall survival (OS), which was measured from the date of CRS until death attributable to any cause or the date of the last documented follow-up contact. Disease-free survival (DFS) served as the key secondary endpoint and was calculated from the date of surgery to the earliest occurrence of confirmed disease recurrence or death from any cause. Evidence of recurrence was established through radiological assessment via CT, MRI, or PET-CT, or through pathological confirmation when endoscopic or clinical findings raised concern. Subjects who remained alive and recurrence-free at the time of data cut-off were censored at the last follow-up. Further secondary outcomes of interest included the incidence of severe postoperative morbidity (Clavien–Dindo grade III or above) and identification of clinicopathological factors independently associated with survival.

### 2.4. Statistical Analysis

Statistical analyses were performed using R software (version 4.4.1; R Foundation for Statistical Computing, Vienna, Austria). Categorical variables are presented as frequencies and percentages and were compared using the chi-square test or Fisher’s exact test, as appropriate. Continuous variables are presented as medians with interquartile ranges (IQR) and were compared using the Mann–Whitney U test or Student’s *t*-test, as appropriate.

Survival curves were estimated using the Kaplan–Meier method and compared between cohorts using the log-rank test. Univariate and multivariate Cox proportional hazards regression analyses were performed to identify the independent prognostic factors for OS. Variables with *p* < 0.05 in univariate analysis were entered into the multivariate model; cohort period (2011–2013 vs. 2014–2019) was included in the multivariate model regardless of its univariate *p*-value, as it represented the primary exposure of interest. The IPC type was excluded from the multivariate analysis due to collinearity with the cohort period. The results are presented as hazard ratios (HRs) with 95% confidence intervals (CIs). All statistical tests were two-sided, and *p* < 0.05 was considered statistically significant.

## 3. Results

### 3.1. Baseline Characteristics

A total of 160 patients were included in the final analysis, comprising 42 patients in the early cohort (2011–2013) and 118 patients in the later cohort (2014–2019) (Table 1).

Overall, the baseline clinicopathological characteristics were largely comparable between the two cohorts. The median age was higher in the early cohort (56 vs. 52 years, *p* = 0.014), whereas sex, tumour location, disease timing, preoperative CEA level, extent of peritonectomy, and histological subtype were comparable between the groups.

Preoperative systemic chemotherapy was administered to approximately 30% of the patients in both cohorts (31.0% vs. 26.3%, *p* = 0.702). The disease burden, as reflected by the PCI distribution, was also similar, with the majority of patients presenting with an intermediate PCI (11–20) (50.0% vs. 39.0%, *p* = 0.444). Complete cytoreduction (CCR0–1) was achieved at a comparable rate (76.2% vs. 72.9%, *p* = 0.830).

In contrast, the type of IPC differed markedly between the cohorts (*p* < 0.001), reflecting a shift in institutional practice. EPIC was predominantly used in the early cohort (85.7%), whereas HIPEC was the primary modality in the later cohort (70.3%), with an additional 23.7% of patients receiving a combination of HIPEC and EPIC.

Postoperative outcomes were generally similar between the groups. The overall 30-day complication rate did not differ significantly between the groups (35.7% vs. 40.7%, *p* = 0.703); however, Grade IIIa complications were more frequent in the later cohort (31.4% vs. 19.0%, *p* = 0.042). The median hospital stay was shorter in the later cohort (23 vs. 29 days), although the difference was not statistically significant (*p* = 0.752). Adjuvant systemic chemotherapy was administered to most patients in both cohorts (88.1% vs. 87.3%; *p* = 1.000).

### 3.2. Survival Outcomes

**Figure 2:**
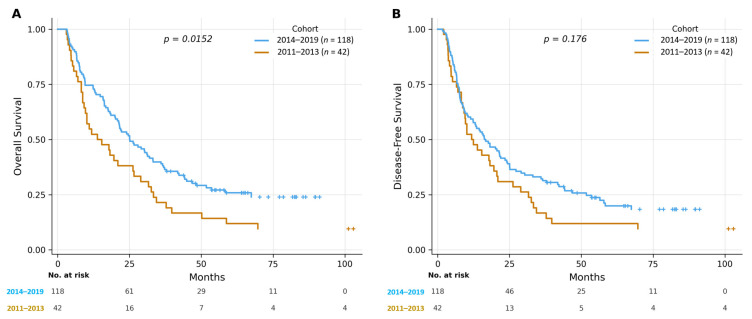
(**A**). Kaplan–Meier curves for overall survival (OS) stratified by cohort period (2011–2013 vs. 2014–2019). (**B**). Kaplan–Meier curves for disease-free survival (DFS) stratified by cohort period (2011–2013 vs. 2014–2019).

Over a median follow-up of 22.1 months (range 3.0–108.1 months), the later cohort demonstrated significantly longer follow-up than the early cohort (25.1 vs. 14.6 months), reflecting the difference in study period duration. The Kaplan–Meier survival curves are illustrated in Figure 2, and the survival outcomes are summarised in Table 2.

The later cohort showed significantly improved overall survival compared to the early cohort (log-rank *p* = 0.015). Median OS was 25.1 months in the later cohort versus 13.8 months in the early cohort, with 5-year OS rates of 25.9% and 11.9%, respectively. The median and 5-year OS rates of the entire cohort were 22.1 months and 22.0%, respectively.

With respect to DFS, the later cohort showed a trend toward improvement, although the difference was not statistically significant (log-rank *p* = 0.176). The median DFS was 16.7 months in the later cohort versus 11.8 months in the early cohort, with 5-year DFS rates of 20.0% and 11.9%, respectively. The overall median DFS was 15.8 months, with a 5-year DFS rate of 18.1%.

### 3.3. Prognostic Factors for Overall Survival

Univariate and multivariate Cox regression analyses are summarized in Table 3.

In univariate analysis, several variables were associated with OS, including cohort period, preoperative systemic chemotherapy, extent of peritonectomy, PCI grade, CCR grade, IPC type, and preoperative CEA level.

Multivariate analysis identified five independent prognostic factors for OS. The later cohort was associated with improved survival (HR 0.63, 95% CI 0.42–0.96, *p* = 0.029). Tumour location remained independently associated with OS (overall *p* = 0.001), with both right colon (HR 3.29, *p* < 0.001) and left colon/rectum (HR 2.62, *p* = 0.003) demonstrating worse survival than appendiceal tumours. Preoperative systemic chemotherapy was associated with reduced mortality (HR 0.54, 95% CI 0.34–0.86, *p* = 0.009), whereas elevated preoperative CEA (≥7 ng/mL) predicted worse survival (HR 1.66, 95% CI 1.11–2.47, *p* = 0.013).

Incomplete cytoreduction (CCR2–3) emerged as the strongest independent predictor of poor survival, with a markedly increased hazard compared with CCR0–1 (HR 4.51, 95% CI 2.73–7.44, *p* < 0.001).

Although the PCI grade was significant in the univariate analysis, it did not retain significance after adjustment (*p* = 0.106), suggesting that its prognostic impact may be mediated through the completeness of cytoreduction. The extent of peritonectomy and postoperative complications were not independently associated with OS.

## 4. Discussion

This study evaluated the impact of accumulated institutional experience on the outcomes of CRS with IPC for colorectal peritoneal metastases by comparing early (2011–2013) and later (2014–2019) patient cohorts at a single high-volume centre. The later cohort demonstrated significantly improved OS compared with the early cohort (median OS 25.1 vs. 13.8 months; 5-year OS 25.9% vs. 11.9%, *p* = 0.015), and cohort period was confirmed as an independent prognostic factor on multivariate analysis (HR 0.63, *p* = 0.029). Notably, this survival improvement was observed despite comparable baseline characteristics and disease burden between the two groups, suggesting that the evolution of treatment strategy—most prominently, the institutional transition from EPIC to HIPEC as the primary IPC modality—and refinements in perioperative care contributed meaningfully to the improved oncological outcomes.

CRS combined with IPC for colorectal peritoneal metastases is a highly complex procedure associated with substantial morbidity, and accumulating evidence suggests that it should be performed at experienced high-volume centres [23]. Previous studies have demonstrated that patients treated at such centres achieve lower operative mortality and improved survival outcomes [24]. These benefits are likely attributable not only to refinements in surgical techniques but also to more stringent patient selection, standardised perioperative management, and the adoption of evolving treatment strategies. In the present study, despite the comparable clinicopathological characteristics and disease burden between the two cohorts, the later cohort showed a significant improvement in OS. The observed survival benefit possibly reflects institutional evolution in treatment strategy rather than a mere effect of time. Notably, there was a transition from EPIC to HIPEC in the later cohort. Although the retrospective nature of this study precludes the establishment of a causal relationship between the adoption of HIPEC and improved outcomes, a 90 min mitomycin C–based HIPEC may enhance cytotoxic effects against residual microscopic disease by increasing the intraperitoneal drug concentration and exploiting the direct cytotoxic synergy between hyperthermia and mitomycin C [25]. Therefore, its contribution to the observed improvement in survival cannot be excluded.

In the multivariate analysis, the CCR score was identified as the strongest independent prognostic factor, with incomplete cytoreduction (CCR2–3) associated with a 4.51-fold higher risk of mortality than complete cytoreduction (CCR0–1) (*p* < 0.001). This finding reaffirms that achieving complete cytoreduction is a key determinant of survival in patients undergoing CRS for IPC [26]. Although PCI was significant in univariate analysis, it was not significant in the multivariate model (*p* = 0.106), suggesting that its effect on survival may be mediated through the likelihood of achieving complete cytoreduction. However, resectability cannot be determined using PCI alone. In practice, the feasibility of complete cytoreduction depends not only on the overall tumour burden, but also on the anatomical distribution of the disease. The involvement of unresectable structures may preclude complete cytoreduction, even in patients with a low PCI, whereas complete cytoreduction may still be achievable in selected patients with a high PCI if the disease is confined to anatomically resectable regions. Therefore, PCI alone may not adequately predict the likelihood of achieving CCR and a comprehensive assessment that incorporates both disease distribution and anatomical considerations is required [27]. Patients who did not receive preoperative systemic chemotherapy had a lower risk of mortality (HR 0.55, *p* = 0.009). This finding likely reflects selection bias, as patients referred after prolonged palliative chemotherapy at outside institutions may have had a poorer performance status or developed treatment resistance. These results underscore the importance of early referral to specialised centres after a diagnosis of peritoneal metastases [28].

In this study, the overall 30-day complication rate did not differ significantly between the two cohorts (35.7% vs. 40.7%, *p* = 0.703); however, the distribution of complication severity differed significantly. The proportion of Clavien–Dindo grade IIIa complications increased in the later cohort (19.0% vs. 31.4%). This increase appears to be primarily attributable to a change in the institutional protocol, whereby postoperative pleural effusion following diaphragmatic peritonectomy or repair was managed with selective drainage based on postoperative imaging rather than routine intraoperative chest tube placement; therefore, it is unlikely to reflect a true worsening of clinical outcomes. More importantly, despite an increase in the number of patients with more advanced disease and the performance of more extensive procedures in the later cohort, there was a trend toward a reduction in fatal complications. This pattern is further supported by a separate analysis conducted at our institution. As reported by Jo et al., comparing 2012–2013 with 2022, the proportion of patients with PCI grade III increased (29.6% to 38.1%), whereas the rate of incomplete cytoreduction (CCR2–3) decreased (35.2% to 26.2%). During the same period, the 30-day mortality rate decreased from 7.4% to 2.4%. Although the incidence of major complications (Clavien–Dindo grade III/IV) increased (22.2% to 45.2%), the failure-to-rescue rate decreased markedly from 33.3% to 5.3% [29]. These findings suggest that increasing institutional experience at high-volume centres may not only reduce mortality but also improve the ability to effectively manage severe complications once they occur, consistent with previous reports [30].

This study had several limitations. First, as this was a retrospective, single-centre study, the possibility of selection bias cannot be excluded. The patient selection criteria and surgical indications may have evolved over the study period, which could have influenced the comparisons between the two cohorts. Moreover, the single-centre nature of this study may limit the generalisability of the findings. Second, it is difficult to clearly distinguish whether the survival improvement observed in the later cohort was attributable to the introduction of HIPEC or to other factors such as accumulated surgical experience, improvements in perioperative management, or advances in systemic chemotherapy over time. Therefore, prospective studies are needed to clarify the independent effects of HIPEC. Third, DFS did not differ significantly between the two cohorts (*p* = 0.176). This may be partly explained by the difference in median follow-up duration (14.6 vs. 25.1 months), and the relatively shorter follow-up in the later cohort may have limited the ability to capture long-term differences in DFS. Longer follow-up and prospective cohort studies with larger sample sizes are required to validate these findings.

## 5. Conclusions

This study suggests that accumulated institutional experience and evolving treatment strategies were associated with improved outcomes in patients undergoing CRS with IPC for colorectal and appendiceal peritoneal metastases at a single high-volume centre. Early referral to a specialised centre and achievement of complete cytoreduction are critical. Structured perioperative management, together with appropriate selection of IPC strategies, may contribute to improved survival. Further prospective multicentre studies are required to clarify the independent effects of these factors.

## Figures and Tables

**Table 1:** Baseline characteristics of the patients.

Variable	2011–2013 (*n* = 42)	2014–2019 (*n* = 118)	*p*-Value
Age (years)	56 [51–62]	52 [44–60]	0.014
**Sex**			
Female	22 (52.4)	63 (53.4)	1.000
Male	20 (47.6)	55 (46.6)	
**Tumor location**			
Appendix	3 (7.1)	20 (16.9)	0.390
Right colon	17 (40.5)	36 (30.5)	
Left colon	19 (45.2)	53 (44.9)	
Rectum	3 (7.1)	9 (7.6)	
**Timing of peritoneal metastasis**			
Metachronous	20 (47.6)	69 (58.5)	0.301
Synchronous	22 (52.4)	49 (41.5)	
**Preoperative chemotherapy**			
Yes	13 (31.0)	31 (26.3)	0.702
No	29 (69.0)	87 (73.7)	
**Preoperative CEA**			
<7 ng/mL	19 (45.2)	58 (49.2)	0.633
≥7 ng/mL	21 (50.0)	55 (46.6)	
Unknown	2 (4.8)	7 (5.9)	
**Extent of peritonectomy**			
Total peritonectomy	15 (35.7)	40 (33.9)	0.403
Partial peritonectomy	24 (57.1)	60 (50.8)	
None	3 (7.1)	18 (15.3)	
**Peritoneal cancer index score**			
1–9	13 (31.0)	42 (35.6)	0.444
10–20	21 (50.0)	46 (39.0)	
21–39	8 (19.0)	30 (25.4)	
**Completeness of cytoreduction score**			
CCR 0–1	32 (76.2)	86 (72.9)	0.830
CCR 2–3	10 (23.8)	32 (27.1)	
**Type of intraperitoneal chemotherapy**			
HIPEC	2 (4.8)	83 (70.3)	< 0.001
EPIC	36 (85.7)	7 (5.9)	
Both	4 (9.5)	28 (23.7)	
**Histological type**			
WD/MD	23 (54.8)	56 (47.5)	0.633
PD/SRC/MUC	16 (38.1)	55 (46.6)	
Others	3 (7.1)	7 (5.9)	
Hospital stay (days)	29 [20–44]	23 [17–32]	0.752
**Postoperative complications (30-day)**			
Any complication	15 (35.7)	48 (40.7)	0.703
**Clavien–Dindo classification**			
None	27 (64.3)	67 (56.8)	0.042
Grade I	0 (0.0)	4 (3.4)	
Grade II	3 (7.1)	1 (0.8)	
Grade IIIa	8 (19.0)	37 (31.4)	
Grade IIIb	1 (2.4)	7 (5.9)	
Grade IVa	2 (4.8)	2 (1.7)	
Grade IVb	1 (2.4)	0 (0.0)	
**Adjuvant chemotherapy**			
Yes	37 (88.1)	89 (87.3)	1.000
No	5 (11.9)	13 (11.0)	

Data are presented as the median [interquartile range] or *n* (%). CEA, carcinoembryonic antigen; HIPEC, hyperthermic intraperitoneal chemotherapy; EPIC, early postoperative intraperitoneal chemotherapy; WD, well-differentiated; MD, moderately differentiated; PD, poorly differentiated; SRC, signet ring cell carcinoma; MUC, mucinous carcinoma; CCR, completeness of cytoreduction.

**Table 2:** Survival outcomes stratified by cohort period.

Outcome	Early Cohort (*n* = 42)	Later Cohort (*n* = 118)	Total (*n* = 160)	*p*-Value
**OS**				
Median OS (months)	13.8	25.1	22.1	0.015
5-year OS rate (%)	11.9	25.9	22.0	
**DFS**				
Median DFS (months)	11.8	16.7	15.8	0.176
5-year DFS rate (%)	11.9	20.0	18.1	

*p*-values were derived using log-rank tests. OS, overall survival; DFS, disease-free survival.

**Table 3:** Univariate and multivariate Cox proportional hazards analyses of prognostic factors associated with overall survival.

Variable	Univariate Analysis		Multivariate Analysis	
	HR (95% CI)	*p*-Value	HR (95% CI)	*p*-Value
**Cohort period**				
2011–2013 (Ref.)	Reference		Reference	
2014–2019	0.63 (0.43–0.92)	0.020	0.63 (0.42–0.96)	0.029
**Sex**				
Female (Ref.)	Reference		–	
Male	0.76 (0.53–1.08)	0.129	–	–
**Tumor location**				
Appendix (Ref.)	Reference		Reference	
Right colon	1.75 (0.94–3.25)	0.079	3.29 (1.64–6.59)	<0.001
Left colon and rectum	1.92 (1.06–3.47)	0.032	2.62 (1.37–4.98)	0.003
Overall		0.070		0.001
**Timing of peritoneal metastasis**				
Metachronous (Ref.)	Reference		–	
Synchronous	0.91 (0.64–1.29)	0.582	–	–
**Preoperative chemotherapy**				
Yes (Ref.)	Reference		Reference	
No	0.55 (0.36–0.86)	0.005	0.54 (0.34–0.86)	0.009
**Extent of peritonectomy**				
None (Ref.)	Reference		Reference	
Partial	0.83 (0.48–1.45)	0.519	1.04 (0.58–1.86)	0.903
Total	1.40 (0.79–2.47)	0.249	1.43 (0.75–2.73)	0.284
Overall		0.034		0.359
**Peritoneal cancer index**				
1–9 (Ref.)	Reference		Reference	
10–20	2.14 (1.41–3.26)	<0.001	1.64 (1.01–2.68)	0.046
>20	2.72 (1.67–4.41)	<0.001	1.77 (0.95–3.27)	0.071
Overall		<0.001		0.106
**Completeness of cytoreduction score**				
CCR 0–1 (Ref.)	Reference		Reference	
CCR 2–3	3.42 (2.31–5.07)	<0.001	4.51 (2.73–7.44)	< 0.001
**Type of intraperitoneal chemotherapy †**				
HIPEC (Ref.)	Reference		–	
EPIC	1.39 (0.92–2.10)	0.121	–	–
Both	2.56 (1.62–4.03)	<0.001	–	–
Overall		<0.001	Excluded (collinear with cohort period)	
**Histological type**				
WD/MD (Ref.)	Reference		–	
PD/SRC/MUC	1.32 (0.93–1.87)	0.125	–	–
**Preoperative CEA**				
<7 ng/mL (Ref.)	Reference		Reference	
≥7 ng/mL	1.81 (1.25–2.63)	0.002	1.66 (1.11–2.47)	0.013
Unknown	0.84 (0.44–1.60)	0.600	0.75 (0.38–1.48)	0.410
Overall		0.003		0.012
**Postoperative complication (30-day)**				
No (Ref.)	Reference		–	
Yes	1.39 (0.97–1.99)	0.075	–	–

Variables with *p* < 0.05 in univariate analysis were entered into multivariate analysis; the cohort period was included regardless of the *p*-value. † Type of intraperitoneal chemotherapy was excluded from the multivariate analysis due to collinearity with the cohort period (HIPEC predominated in 2014–2019 vs. EPIC in 2011–2013). HR, hazard ratio; CI, confidence interval; Ref., reference; CCR, completeness of cytoreduction; CEA, carcinoembryonic antigen; HIPEC, hyperthermic intraperitoneal chemotherapy; EPIC, early postoperative intraperitoneal chemotherapy; WD, well-differentiated; MD, moderately differentiated; PD, poorly differentiated; SRC, signet ring cell carcinoma; MUC, mucinous carcinoma.

## Data Availability

All data generated during the course of this study will be made available upon request from the corresponding author.

## Abbreviations

The following abbreviations are used in this manuscript:

CA19-9	cancer antigen 19-9
CEA	carcinoembryonic antigen
CCR	Completeness of Cytoreduction
CRS	cytoreductive surgery
DFS	disease-free survival
ECOG	Eastern Cooperative Oncology Group
EPIC	early postoperative intraperitoneal chemotherapy
HIPEC	hyperthermic intraperitoneal chemotherapy
IPC	intraperitoneal chemotherapy
MRI	Magnetic resonance imaging
OS	overall survival
PCI	Peritoneal Cancer Index
